# Gait Pattern Alterations during Walking, Texting and Walking and Texting during Cognitively Distractive Tasks while Negotiating Common Pedestrian Obstacles

**DOI:** 10.1371/journal.pone.0133281

**Published:** 2015-07-29

**Authors:** Sammy Licence, Robynne Smith, Miranda P. McGuigan, Conrad P. Earnest

**Affiliations:** 1 The University of Bath, Department for Health, Bath, Somerset, United Kingdom; 2 Texas A&M University, Department of Health & Kinesiology, College Station, Texas, United States of America; Ludwig-Maximilian University, GERMANY

## Abstract

**Objectives:**

Mobile phone texting is a common daily occurrence with a paucity of research examining corresponding gait characteristics. To date, most studies have participants walk in a straight line vs. overcoming barriers and obstacles that occur during regular walking. The aim of our study is to examine the effect of mobile phone texting during periods of cognitive distraction while walking and negotiating barriers synonymous with pedestrian traffic.

**Methods:**

Thirty participants (18-50y) completed three randomized, counter-balanced walking tasks over a course during: (1) normal walking (control), (2) texting and walking, and (3) texting and walking whilst being cognitively distraction via a standard mathematical test performed while negotiating the obstacle course. We analyzed gait characteristics during course negotiation using a 3-dimensional motion analysis system and a general linear model and Dunnet-Hsu post-hoc procedure the normal walking condition to assess gait characteristic differences. Primary outcomes included the overall time to complete the course time and barrier contact. Secondary outcomes included obstacle clearance height, step frequency, step time, double support phase and lateral deviation.

**Results:**

Participants took significantly longer (mean ± SD) to complete the course while texting (24.96±4.20 sec) and during cognitive distraction COG (24.09±3.36 sec) vs. normal walking (19.32±2.28 sec; *all*, P<0.001). No significant differences were noted for barrier contacts (P = 0.28). Step frequency, step time, double support phase and lateral deviation all increased in duration during the texting and cognitive distraction trial. Texting and being cognitively distracted also increased obstacle clearance versus the walking condition (*all*, P<0.02).

**Conclusions:**

Texting while walking and/or being cognitively distracted significantly affect gait characteristics concordant to mobile phone usage resulting in a more cautious gate pattern. Future research should also examine a similar study in older participants who may be at a greater risk of tripping with such walking deviations.

## Introduction

Mobile and Smartphone ownership increased internationally as has the individual use of texting, SMS messaging, Internet use, social media access and emailing. For simplicity we will use “text” to denote the various forms of Internet communication functions. While text growth facilitates communication it also increases the risk of distraction during walking, which may in turn carry with it an increased risk for tripping, collision or secondary injuries to other pedestrians attempting to avoid those who are texting and deviating from a normal path of ambulation [1–3]. It has been proffered that these phenomena are due to inattentional blindness and situational awareness [4]. While the risk associated with texting has been documented for during driving, the potential hazards associated with walking are not as well established [5–7].

Few studies have investigated walking and texting and fewer yet the simultaneous negotiation of an obstacle course [8]. This is an important consideration as “real life” requires the user to look away from pathway obstacles in order to text [9]. As such, texting while walking disrupts gait speed, potentially increasing road crossing time and riskier road crossing behaviors, possibly increasing tripping and accident risk [10–13]. While most studies use fairly simplistic models, we are unaware of research using more circuitous obstacle courses designed and built to represent common pedestrian obstacles such as curbs, people, steps, etc. The primary aim of our study is to examine the effect texting on gait characteristics while negotiating common pedestrian obstacles. We hypothesize that texting will affect normal gait characteristics and increase barrier contacts, a surrogate for tripping, while negotiating steps, ramps and obstacles representing pedestrian traffic.

## Materials and Methods

### Participants

We recruited thirty participants (18 females; 18–50 years) to take part in our study approved by the University of Bath Department for Health Ethics Advisory Panel. We included only participants who owned their own mobile phone for more than one month and excluded candidates taking medications that may cause dizziness. All participants signed an informed consent outlining the study aims and procedures. Subsequently, participants completed questionnaires regarding their current mobile phone use and a physical-activity readiness questionnaire [14].

### Experimental Procedures

Before initiating formal testing procedures, participants completed a familiarization session of the obstacle course under each testing condition: (1) walking with no distraction (WLK, control), (2) responding to standardized texting questions on their own phone (TXT) and (3) completing a mental mathematics quiz (AB Math Lite 5.3) on an iPhone (Apple, Cupertino, CA US phone (COG). All participants were instructed not to look at either the messages or the mental mathematics quiz until they began walking. All walking conditions were administered in a randomized, counter-balanced manner.

**Fig 1:**
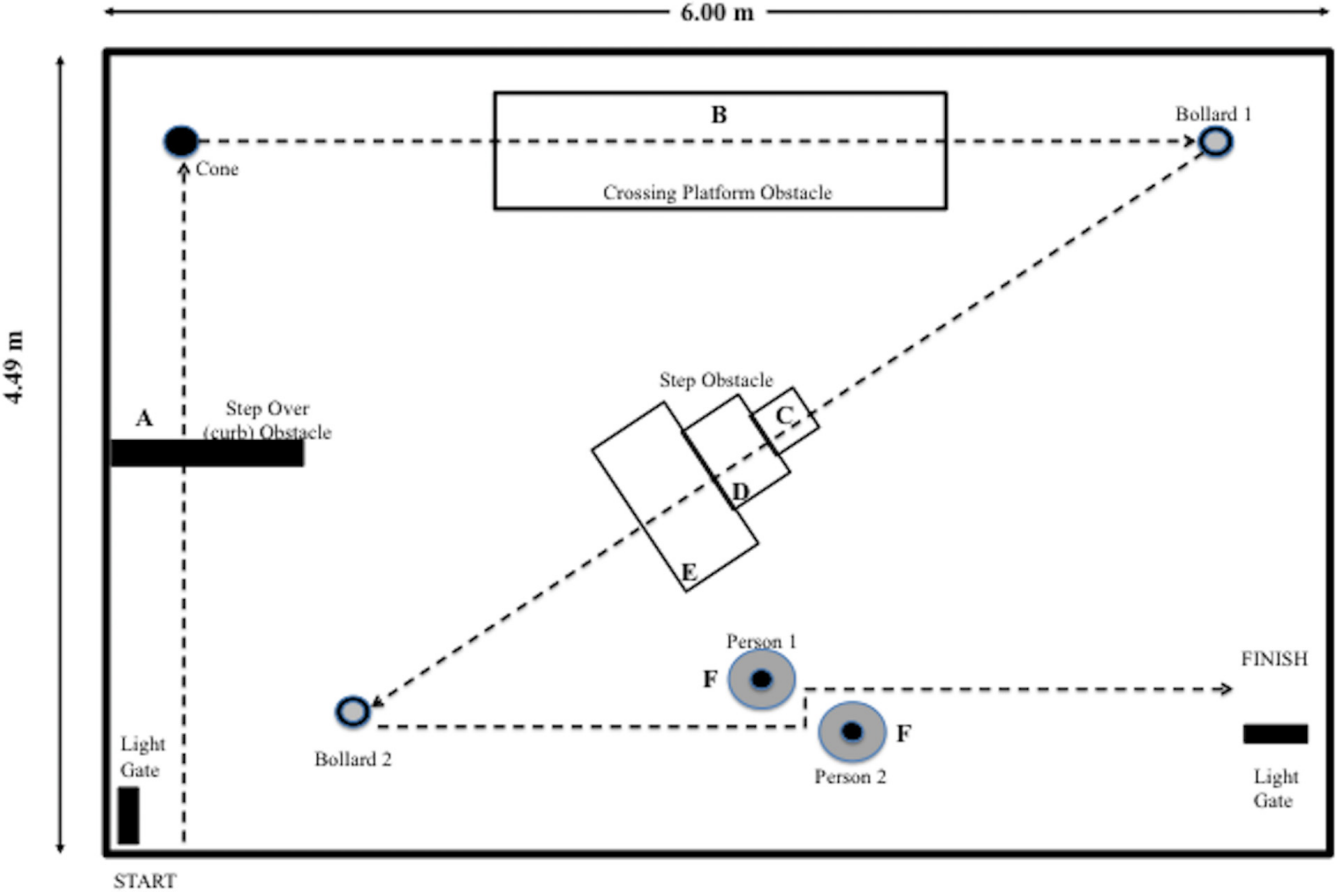
Schematic and dimensional representation of obstacle course obstacles. (**A**) Step Over Curb (Length = 0.105 m; width = 0.720 m; height 0.105 m), (**B**) Crossing Platform (Length = 2.030 m; width = 0.750 m; height = 0.092 m), (**C**) Step Obstacle: (Step 1; Length = 0.400 m; width = 0.400 m; height = 0.205 m, (**D**) Step 2; Length = 0.500 m; width = 0.500 m; height = 0.300 m), (**E**) Step 3; (Length = 0.585 m; width = 0.910 m; height = 0.110 m), (**F**) Model people (Length = 0.420 m; width = 0.530 m; height = 1.880 m).

The obstacle course was designed to mimic obstacles one would encounter in everyday life (**Fig 1**) and consisted of seven obstacles designed and based on fieldwork within the City Centre of Bath, UK: (1) An obstacle resembling a curb to step over, (2) A platform to step-on, step-off platform, (3) A set of uneven steps, (4–5) Two traffic bollards to step around and, (6–7) Two dummies of sufficient height representing model people for participants to step around.

We used a 3D optical motion analysis system (Qualisys, Sweden), with 13 Oqus 4 cameras set-up to collect kinematic data whilst the participants negotiated the obstacle course in each of the conditions. Six reflective markers were attached to each participant using double sided tape: two on each shoe (n = 4), one approximately over the 5th metatarsal-phalangeal (MTP) joint and one on the back of the heel, and two on an elasticated band worn around the pelvis. The two pelvis markers were positioned on either side of the spine, approximately over the posterior superior iliac spines. The elasticated band was used to provide a tight fitting surface to which to attach the pelvis markers as the participants undertook the testing in their everyday clothes. We also placed photocell light gates (Powertimer 1.0, Newtest, Finland) at the start and finish of the course to measure time to complete the course. Reflective markers were placed on every obstacle on the corners of the front edge of each obstacle except for the two model people. The 3D positions of the markers on the participant and the markers on the obstacles were determined at 100Hz. From these data we calculated the velocity of the participant and various step characteristics as they negotiated the course (**Fig 1**). Data from Qualisys was imported into an Excel spreadsheet in order to perform requisite calculations. No filtering was applied or data adjusted we only looked at marker displacement of the markers and did not investigate velocity or acceleration.

### Step Characteristics

To calculate step characteristics we determined the timings of heel strike and toe-off using an algorithm developed by Zeni Jr. et al. (2008). [15] This entailed finding the coordinates of the mid-point of the pelvis markers and subtracting this from the coordinates of heel and 5th MTP markers. Heel strike was defined as the point at which the heel was at a maximum distance in front of the hip and toe-off as the point at which the 5th MTP was at a maximum distance behind the hip. Based upon the timings and positions of heel strike and toe off of each step the following parameters were determined and where appropriate a mean value calculated over the course:

- *Step count*: the number of steps taken for the obstacle course
- *Step time*: the time between the heel strike of one foot and the heel strike of the contralateral foot
- *Step frequency*: the number of steps per second
- *Step length*: the distance between the position of the heel marker at heel strike of one foot and the position of the heel marker at heel strike of the contralateral foot
- *Double support phase*: the time between second foot heel strike and first foot toe off or the time during which both feet were in contact with the ground
- *Barrier clearance*: the difference between the 5^th^ MTP marker vertical coordinate and the barrier vertical coordinate at the time the 5th MTP joint arrived at the obstacle.

We also performed a sub-analysis to examine some of these gait characteristics during the approach and negotiation of the obstacles. *Stair approach* is defined as each respective gait characteristic observed in the 1.5 m immediate prior to negotiating the stairs. *Stair ascent* is defined as each respective gait characteristic observed in the ascending the stairs. *Platform traverse* is each respective gait characteristic observed while traversing the platform.

In addition to the outcome measures defined above, the deviation from a straight path was investigated during the first two sides of the obstacle course. This part of the course was chosen as it involved two clearly defined straight channels with a 90° turn between them (Fig 1). The length of the straight-line path of the first and second sides of the course was determined from the co-ordinates of the centre of the beginning and the end of the path for these two sides (as defined by cones and markers on the ground). The length of the path travelled by the participants was defined as the cumulative displacement of the centre of the pelvis markers along the first and second sides of the course. This was calculated by determining the centre point of the two pelvis markers at each time point, calculating the displacement of this point between each time point and summing these from the beginning of the course until the participant reached the end of the second side. The deviation from walking a straight path was determined by subtracting the straight path distance from the actual path travelled.

### Statistical Analysis

We examined our data using a general linear model covaried for the control/WLK condition using SPSS (v21). Data sphericity was accounted for via a Greenhouse-Geisser correction. Post-hoc analyses were performed using a Dunnett-HSU analysis to compare TXT and COG to the WLK condition. We also performed Pearson correlations between various gait characteristics and barrier contact in an effort to associate potential gait characteristics and tripping risk. All data is presented as mean ± standard deviation (SD) or 95% confidence intervals where appropriate. Statistical significance was established at P < 0.05. The effect size is presented as the partial eta squared for each analysis.

## Results and Discussion

### Course Performance Characteristics

**Fig 2:**
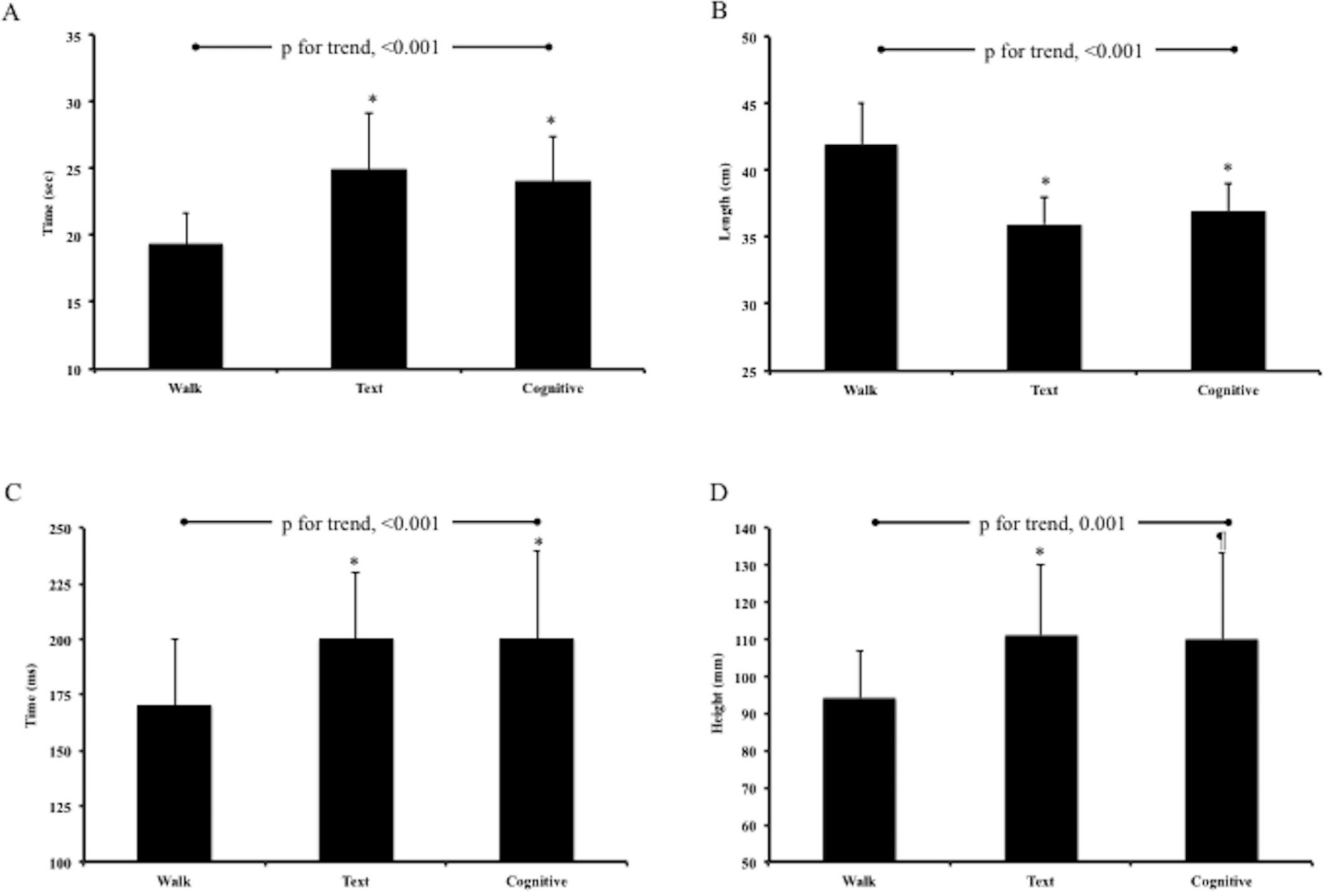
Data represent mean and 95% confidence interval information for Course Time (Panel A), Step Length (Panel B), Double Support phase of walking (Panel C) and Obstacle Clearance Height for course barriers (Panel D). Statistical notations are: * 0.001 and ¶ 0.02.

We have presented the overall findings for negotiating the complete course in **Table 1**. Overall, we found that it took significantly longer to complete the course due to slower walking speeds under the TXT and COG conditions vs. WLK (*all*, P<0.001, **Fig 2A**). In support of these observations, participants exhibited significantly shorter step lengths (**Fig 2B**), lower step frequencies, longer double support phases (**Fig 2C**), and greater obstacle clearance heights while TXT and COG vs. WLK (all, P<0.001, **Fig 2D**). While the COG condition was significantly different to TXT for walking speed (P<0.02) and step frequency (P<0.04), no other significant differences were noted between the TXT and COG conditions. Despite these alterations in gait characteristics we did not observe any changes in barrier contacts during any treatment condition (P = 0.10).

**Table 1:** Course performance characteristics of participants negotiating obstacle course.

	Distraction Challenge	Mean	SD	Significance vs. WLK	Partial Eta Squared
Course Time (sec)	Walk	19.32	2.3		
	Text	24.96	4.2	< 0.001	
	Cognitive	24.09	3.4	< 0.001	0.79
Walk Speed (m/sec)	Walk	0.78	0.1		
	Text	0.61	0.1	< 0.001	
	Cognitive ^a^	0.63	0.1	< 0.001	0.88
Lateral Deviation	Walk	2.71	0.3		
from Straight Path	Text	2.76	0.3	NS	
	Cognitive	2.77	0.6	NS	0.04
Barrier Contact (n)	Walk	0.13	0.4		
	Text	0.23	0.4	NS	
	Cognitive	0.10	0.3	NS	0.10
Step Length (cm)	Walk	42.00	30.0		
	Text	36.00	20.0	< 0.001	
	Cognitive	37.00	20.0	< 0.001	0.87
Step Frequency (steps/sec)	Walk	1.84	0.2		
	Text	1.67	0.2	< 0.001	
	Cognitive ^b^	1.71	0.2	< 0.001	0.62
Double Support Phase (sec)	Walk	170.00	30.0		
	Text	200.00	30.0	< 0.001	
	Cognitive	200.00	40.0	< 0.001	0.26
Obstacle Clearance Height (mm)	Walk	94.00	13.0		
	Text	111.00	19.0	< 0.001	
	Cognitive	110.00	23.0	<0.02	0.40

a = Significant vs. Text = 0.015.

b = Significant vs. Text = 0.035.

### Performance Characteristics for Individual Obstacles

We have presented the findings for negotiating each individual obstacle in **Table 2**.

**Table 2:** Performance characteristics for individual obstacles.

			Mean	SD	Significance vs. WLK	Partial Eta Squared
Time (ms)	Stair	Walk	550.00	60.0		
	Approach	Text	580.00	90.0	0.074	
		Cognitive	590.00	70.0	0.004	0.267
	Platform	Walk	630.00	60.0		
	Traverse	Text	710.00	110.0	< 0.001	
		Cognitive	710.00	100.0	< 0.001	0.453
	Stair	Walk	480.00	130.0		
	Ascent	Text	570.00	90.0	0.000	
		Cognitive	550.00	70.0	0.008	0.414
Step count (n)	Stair	Walk	1.24	0.4		
	Approach	Text	1.43	0.5		
		Cognitive	1.38	0.5		0.140
	Platform	Walk	1.07	0.3		
	Traverse	Text	1.40	0.5	0.004	
		Cognitive	1.24	0.4	0.060	0.270
	Stair	Walk	1.10	0.3		
	Ascent	Text	1.53	0.5	< 0.001	
		Cognitive	1.34	0.5	< 0.001	0.400
Step Frequency (steps/sec)	Stair	Walk	1.83	0.2		
	Approach	Text	1.89	0.5	NS	
		Cognitive	1.83	0.5	NS	0.001
	Platform	Walk	1.61	0.2		
	Traverse	Text	1.55	0.5	NS	
		Cognitive	1.44	0.2	< 0.001	0.556
	Stair	Walk	2.17	0.6		
	Ascent	Text	1.80	0.4	0.005	
		Cognitive	1.87	0.3	0.014	0.266
Step Length (cm)	Stair	Walk	69.70	17.1		
	Approach	Text	61.20	7.7	0.011	
		Cognitive	59.90	10.7	0.004	0.268
	Platform	Walk	72.40	7.0		
	Traverse	Text	58.40	15.7	0.001	
		Cognitive	64.60	9.4	0.001	0.486
	Stair	Walk	54.40	23.8		
	Ascent	Text	48.70	18.2	NS	
		Cognitive	50.80	14.6	NS	0.087
Double Support Phase (ms)	Stair	Walk	150.00	30.0		
	Approach	Text	170.00	40.0	0.004	
		Cognitive	170.00	20.0	< 0.001	0.483
	Platform	Walk	170.00	70.0		
	Traverse	Text	190.00	40.0	NS	
		Cognitive	200.00	80.0	NS	0.097
	Stair	Walk	220.00	120.0		
	Ascent	Text	290.00	150.0	NS	
		Cognitive	280.00	160.0	<0.001	0.440
Clearance Height (mm)	Stair	Walk	135.00	26.0		
	Approach	Text	159.00	41.0	<0.001	
		Cognitive	148.00	51.0	NS	0.365
	Platform	Walk	65.00	26.0		
	Traverse	Text	65.00	22.0	NS	
		Cognitive	65.00	32.0	NS	0.000
	Stair	Walk	73.00	14.0		
	Ascent	Text	97.00	37.0	0.003	
		Cognitive	99.00	22.0	<0.001	0.615

#### Step Approach

When negotiating the 1.5 m approach to the steps, we did not observe a significant difference to the step count or step frequency on the approach. However, we did observe a significantly greater length of time to the approach for the COG condition and a strong trend for significance during the TXT condition (P = 0.07) due to a significant reduction in step length and double support phase for the TXT (P = 0.011) and COG conditions (P = 0.004). While we also observed a greater step clearance height for the TXT condition (P<0.001), we did no observe a significant for the COG condition.

#### Platform Traverse

Overall, it took longer to negotiate the platform during the TXT and COG conditions (all, P<0.001) vs. WLK with no distraction. This was due, in part, to a greater step count for the TXT condition (P = 0.004) and strong trend for the COG condition (P = 0.06). Other contributing factors for this observation included a shorter step frequency for the COG condition (P<0.001) and shorter step lengths for the TXT and COG condition (*both*, P<0.001). No significant differences were noted for the double support phase or clearance height.

#### Stairs Ascent

Negotiating the stairs took significantly longer during the TXT (P<0.001) and COG conditions P = 0.008). Contributing factors for this observation included a greater step count during the TXT and COG conditions (P<0.001), a greater step frequency during the TXT (P = 0.005) and COG conditions (P = 0.014), and greater clearance heights whilst negotiating the stairs for the TXT (P = 0.003) and COG conditions (P<0.001). No significant differences were noted for step length or the double support phase of stair ascent.

## Discussion

The primary aim of our study was to examine the effect of a TXT and COG challenge on gait characteristics during concomitant walking and mobile phone use. Our primary findings show that TXT and COG significantly shorten step length, reduce step frequency, lengthen double phase support and increase obstacle clearance height. Our secondary outcome analysis also shows a similar pattern alterations relative to each respective obstacle encountered over the course. Specifically, participants altered their approach to stairs, subsequent stair ascent obstacle step clearance height and the negotiation of platforms under the TXT and COG conditions. Thus, we accept our first research hypothesis that TXT and COG significantly alters gait characteristics during walking. While one might infer that these alterations in gait might increase the risk for tripping, our surrogate analysis (i.e., barrier contacts) showed no significant differences between any treatment conditions. Therefore, we reject our hypothesis that TXT and COG would increase the occurrence barrier contact and that the connection between gait alterations, deviations from walking a straight path and tripping becomes tenuous. Our results, in conjunction with others [16–18], suggest that those who walk and text adopt a “protective” gait pattern alteration in order to minimize the risk of potential accidents.

Overall, the adoption of a more conservative locomotion strategy involves a decreased walking velocity and foot placement adjustment before encountering an obstacle [16–18]. Some hypothesize that when faced with a cognitive challenge, posture and gait function are prioritized over cognitive demands, as posture and gait operate at a more subconscious level [19, 20]. Whether the change in gait pattern in our study is conscious or sub-conscious is currently indeterminable; however, the decrease in walking velocity associated with a TXT and COG challenge in our study coupled with previous literature examining other populations examining walking support this hypothesis [11, 16–18, 21, 22]. In essence, when faced with the dual task challenge of walking and texting while undergoing a cognitive challenge, participants decrease their walking speed to avoid accidents [23]. The results from the present study support this mechanism given that participants displayed adaptive changes in gait patterns during the TXT and COG conditions. As it pertains to texting our findings differ to those recently published by Plummer et al (2015) who demonstrated no significant gait alterations using a single and dual task assessment of walking and texting in 32 young adults (18–30 y) [20]. We propose several reasons for these differences.

First, Plummer et al (2015) used a straight course pathway free of obstacles, while our course was circuitous and incorporated common pedestrian obstacles. Second, we used a broader age range (18–50 y), which may have introduced a “familiarity” component as younger individuals are more likely to be familiar with the dual tasking associated with walking whilst texting. Specifically, those individuals under the age of 30, such as those in the Plummer study, likely started using mobile phones. While we attempted to explore an ageing component within our study generally feel that the upper age limit within our study was not sufficient to separate out more robust ageing differences. However, our findings are generally consistent with previous research showing that when time constraints are imposed on participants, older individuals typically take longer or exhibit more cautious gate characteristics than their younger counter parts [24–26]. Though currently absent from the literature a more thorough investigation into the effects of simultaneous texting and walking, as older individuals are more susceptible to tripping (12, 13). While our study does support a greater risk for tripping for the age group we examined, other areas of walking behaviours that warrant attention include slower road crossing time and riskier road crossing behaviors, all of which are associated with an increased tripping and accident risk [10–13]. Others have shown that walking increases ones time spent looking away from obstacles by as much as 400% [9] leading to a phenomenon a known as “inattentional blindness” [27–29]. Citing a more humorous example, Hyman et al. (2010) showed that people using mobile phones were less likely to notice a unicycling clown performing along their walking route [4].

Our study presents for the first time gait characteristics associated with TXT and COG challenges while negotiating a serpentine course designed to emulate common outdoor walking tasks. Though we attempted to examine gender and aging affects by recruiting a more mature population, the age gap was likely insufficient to distinguish more robust obstacle course differences and we are therefore unable to generalize our findings beyond the age limits associated within our study cohort. We also cannot generalize to the potential effects of TXT and COG distractions associated with mobile phone use beyond our laboratory conditions. Our study is strengthened by our use of a crossover design, as well as our incorporation of an unfamiliar phone to decrease any phone-familiarity bias. In retrospect, our study would be enhanced by the use of eye-tracking technology, which would allow for more conclusions to be drawn regarding the attention prioritization encountered during course negotiation.

The present study supports the premise that gait is not a completely automated function and is altered by the addition of TXT and COG tasks. The minimal differences between the TXT and COG conditions in the present study potentially show that even though the conditions could require different cognitive responses, those under the age of 50 y are used to interacting with mobile technology whilst walking. Based on our findings, participants display a more conservative and hesitant gait in response to the dual-tasking situation of COG and TXT. The present study has, however, advanced the relevant field of research via the use of a more sophisticated methodology, which has in turn provided greater insight into the relationship between mobile phone usage gait alterations.

## Supporting Information

S1 Table Raw Data (XLSX)
